# Heterogeneous Periostin Expression in Different Histological Variants of Papillary Thyroid Carcinoma

**DOI:** 10.1155/2017/8701386

**Published:** 2017-12-25

**Authors:** Simona Eliza Giusca, Cornelia Amalinei, Ludmila Lozneanu, Delia Ciobanu Apostol, Elena Corina Andriescu, Alex Scripcariu, Raluca Balan, Elena Roxana Avadanei, Irina-Draga Căruntu

**Affiliations:** ^1^Department of Morphofunctional Sciences I-Histology, Pathology, “Grigore T. Popa” University of Medicine and Pharmacy, Iasi, Romania; ^2^Department of Pathology, Institute of Legal Medicine, Iasi, Romania; ^3^Department of Pathology, “Sf. Spiridon” County Clinical Emergency Hospital, Iasi, Romania

## Abstract

**Background:**

Periostin (PN) epithelial and stromal overexpression in tumor pathology has been studied according to tumor growth, angiogenesis, invasiveness, and metastasis, but a limited number of studies address PN in thyroid tumors.

**Aim:**

Our study aimed to analyze PN expression in different histological variants of PTC and to correlate its expression with the clinicopathological prognostic factors.

**Material and Methods:**

PN expression has been immunohistochemically assessed in 50 cases of PTC (conventional, follicular, oncocytic, macrofollicular, and tall cell variants), in tumor epithelial cells and intratumoral stroma. The association between PN expression and clinicopathological characteristics has been evaluated.

**Results:**

Our results show that PTC presented different patterns of PN immunoreaction, stromal PN being significantly associated with advanced tumor stage and extrathyroidal extension. No correlations were found between PN overexpression in tumor epithelial cells and clinicopathological features, except for specific histological variants, the highest risk of poor outcome being registered for the conventional subtype in comparison to the oncocytic type.

**Conclusions:**

Our study demonstrates differences in PN expression in histological subtypes of PTC. Our results plead in favor of a dominant protumorigenic role of stromal PN, while the action of epithelial PN is less noticeable.

## 1. Introduction

Thyroid cancer represents less than 1% of total number of cancers, but during the last decades its incidence has been continuously growing, showing a dominant involvement of female sex and of young and medium ages [1]. Papillary thyroid carcinoma (PTC) is the most common histological type. Diagnosed in approximate 85% of cases [2], this histological type has a relatively good prognosis, distant metastases, and death being rare events.

Classically, the prognostic assessment of PTC relies, according to the WHO, on the following standard clinical and morphological factors: patients' age and sex, tumor size, histological variant, extrathyroidal extension, completeness of surgical resection, and occurrence of distant metastasis [1].

Tremendous progress has been made by genomics, transcriptomics, and proteomics in all types of cancers, including PTC, resulting in a switch over from traditional clinicopathological prognosis factors to new molecular prognosis markers.

The current trend in thyroid tumor pathology is to improve the grading framework by implementation of new molecular and genetic criteria that could explain the differences between the biological behaviors, quantified by low versus high PTC aggressiveness. Consequently, a large series of molecular markers have been investigated, but none of them has been yet validated and thus they are still considered as candidate prognosis factors. Therefore this issue is remaining a source of heated debate.

As a component of the cellular matrix, periostin (PN) has been recently included in the list of putative prognostic markers. PN is a cellular adhesion molecule, initially identified within the osteoblastic cellular line in mice [3] and named according its identification in periosteum and periodontal ligamentum [4].

PN is secreted by fibroblasts [5–7] and belongs to fasciclin-I family of proteins, functioning in cell-cell and cell-extracellular matrix (ECM) interactions. It is located in fetal and normal adult organs, such as embryonic periosteum, placenta, heart valves, thyroid, adrenal glands, lung, stomach, colon, testicle, prostate, vagina, ovary, breast, and periodontal ligamentum [8–11].

PN epithelial and stromal overexpression in tumor pathology has been studied according to tumor growth, angiogenesis, invasiveness, and metastasis [10–14]. The published data are relatively limited but nevertheless they are supporting PN involvement in tumor progression in different locations, such oral [15] and head and neck carcinomas [16, 17], breast cancer [18–23], ovarian cancer [8, 12, 24–26], prostate cancer [27–30], renal cell carcinoma [31–33], pancreatic carcinoma [34, 35], stomach [36–38], colon [39, 40] and hepatocellular carcinoma [13, 41, 42], non-small-cell lung carcinoma [43–46], malignant pleural mesothelioma [47], neuroblastoma [48], glioblastoma [49–51], and its association with aggressive phenotypes and poor prognosis [7, 10, 13, 14].

To the best of our knowledge, PN expression in thyroid tumors is scarcely reported in the mainstream publications.

Within this context, the present study aims to analyze PN expression in different histological variants of PTC and to correlate its expression with the clinicopathological prognostic factors.

## 2. Material and Method

### 2.1. Patients and Tissues

The study group is comprised of 50 patients diagnosed with PTC in “Sf. Spiridon” County Clinical Emergency Hospital and surgically treated by thyroidectomy with cervical lymph node dissection.

The clinicopathological features have been retrospectively documented from the medical files and included the following data: sex, age (<45 and ≥45 years old, resp.), tumor size, multifocality (number of foci), lymphovascular invasion, extrathyroidal extension (defined as microscopic presence of tumor cells beyond the thyroid capsule, into adipose tissue, skeletal muscle, or sizable vessels and nerves), lymph node metastasis, and tumor stages according to TNM and American Joint Committee on Cancer staging system [52].

All cases have been reassessed by two independent pathologists in order to identify the histological variant of PTC and to confirm the associated thyroid pathology.

The study has been approved by the Ethics Committee of “Grigore T. Popa” University of Medicine and Pharmacy Iasi, complying with the ethical standards of Helsinki declaration that require the patients' informed consent on the usage of their biologic material.

### 2.2. Immunohistochemistry

For each case, a representative paraffin-embedded block has been chosen, and 3 *μ*m sections have been cut and have been displayed on electrostatically charged polylysine-coated slides.

Tissue samples were dewaxed in xylene and rehydrated in 3 baths of alcohol with progressive decreasing concentrations. Heat induced epitope retrieval technique was used for antigen retrieval. The slides were immersed in sodium citrate pH 6 and boiled in water bath at 98°C for 30 minutes. After cooling at room temperature and inhibition of endogenous peroxidase activity, the samples have been incubated with anti-periostin polyclonal antibody (Santa Cruz, Biotechnology Inc., Santa Cruz, USA) dilution 1 : 100, overnight at 4°C. Immunoreaction has been amplified with the suitable secondary and tertiary antibodies of the LSAB-HRP complex (Dako, Carpinteria, USA) and developed with 3,3′-diaminobenzidine tetrahydrochloride chromogen (DakoCytomation, Carpinteria, USA); the positive reaction has been considered in the presence of a brown cytoplasmic stain. Positive and negative controls have been simultaneously run.

### 2.3. Semiquantitative Assessment

PN expression has been separately assessed in tumor epithelial cells and in intratumoral stroma, using adapted semiquantitative scores based on literature reports [25, 53, 54]. The corresponding nontumoral thyroid tissue within each PTC specimen has been constantly evaluated. This step allowed us to establish the basal level of thyroid tissue PN immunoreaction, considering the staining of the follicular cells within these areas as absent or weak (+). We have evaluated the staining intensity in the tumor cellular component – *I* (0 when absent, 1 for weak (+), 2 for moderate (++), and 3 for strong (+++) intensity, resp.) and percentage of positive tumor cells – *P* (0 for <10%, 1 for 10–30%, 2 for 30–60%, and 3 for >60% positive cells, resp.). The final score has been obtained as a sum between *I* and *P*, with a minimum value of 0 and a maximum one of 6. We have considered the values between 0 and 3 as low score (corresponding to PN negative or weak expression) and those between 4 and 6 as high score (revealing a high PN expression). The stromal PN reaction has been quantified as 0 for no staining or less than 5% and 1 for >5% of positive intratumoral stroma, respectively.

### 2.4. Statistical Analysis

Statistical analysis has been performed by GraphPad Prism software package (GraphPad Software, San Diego, CA, USA). The association between PN expression and clinicopathological characteristics has been analyzed by applying the *χ*^2^ test, whereas odds ratios (ORs) using logistic regression have been calculated to assess the correlation between PN and outcome variables for tumor aggressiveness. Statistically significant results have been considered when *p* < 0.05.

## 3. Results

### 3.1. Clinicopathological Characteristics

A predominant female sex was observed in our study group, 41 cases (82.0%), compared to male sex, 9 cases (18.5%). The mean age at diagnosis was 48.24 ± 14.70 years (range 19–76 years), 42.0% (21 patients) being diagnosed at young age, under 45 years. Mean tumor size was 2.18 ± 1.36 cm (range 1.1–7.5 cm). Multifocality was present in 34 cases (68%). We noted lymphovascular invasion in 14 cases (28%), extrathyroidal extension in 23 cases (46.0%), and lymph node metastasis in 7 cases (14%).

Based on TNM and AJCC criteria, the cases were staged as follows: 18 cases (36%), stage I, 6 cases (12%), stage II, 25 cases (50%), stage III, and 1 case (2%), stage IV.

Histologically, there were 10 cases (20%) diagnosed as conventional PTC and 40 cases (80%) as other variants of PTC (follicular, 21 cases (42%), oncocytic, 8 cases (16%), macrofollicular, 7 cases (14%), and tall cell, 4 cases (8%)).

### 3.2. PN Expression

#### 3.2.1. Qualitative Assessment

PN immunopositivity has been noticed in both tumor cells and intratumoral stroma.

PN expression exhibited a predominantly cytoplasmic, perinuclear, finely granular pattern, in tumor cells. The distribution was predominantly homogenous, though some heterogenous areas were focally identified. The reaction intensity was predominantly moderate or strong.

**Figure 1:**
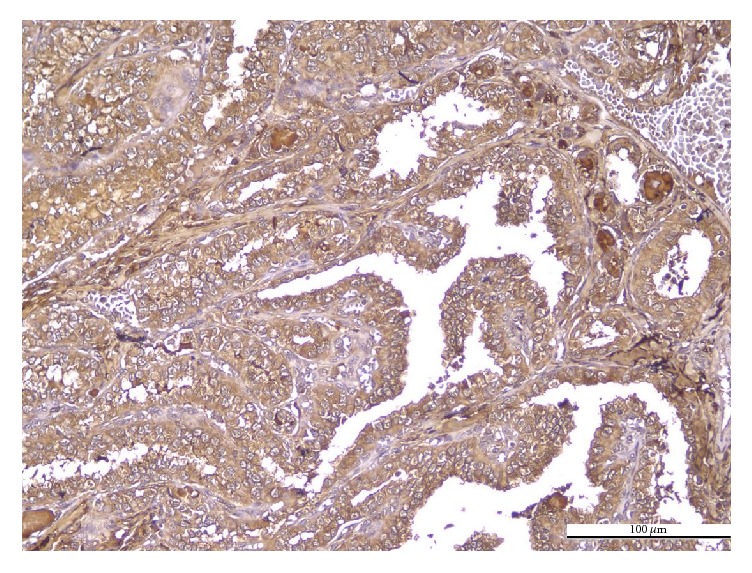
Conventional PTC. Positive PN in tumor cells: diffuse cytoplasmic positive immunoreaction of moderate intensity; negative PN in intratumoral stroma (IHC anti-PN, ×200).

**Figure 2:**
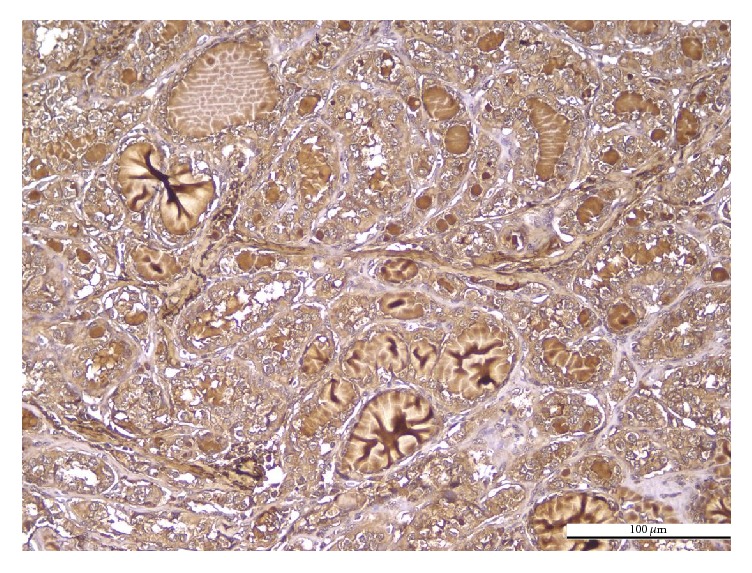
Macrofollicular PTC. Positive PN in tumor cells: diffuse cytoplasmic immunoreaction of moderate intensity; negative PN in intratumoral stroma (IHC anti-PN, ×200).

**Figure 3:**
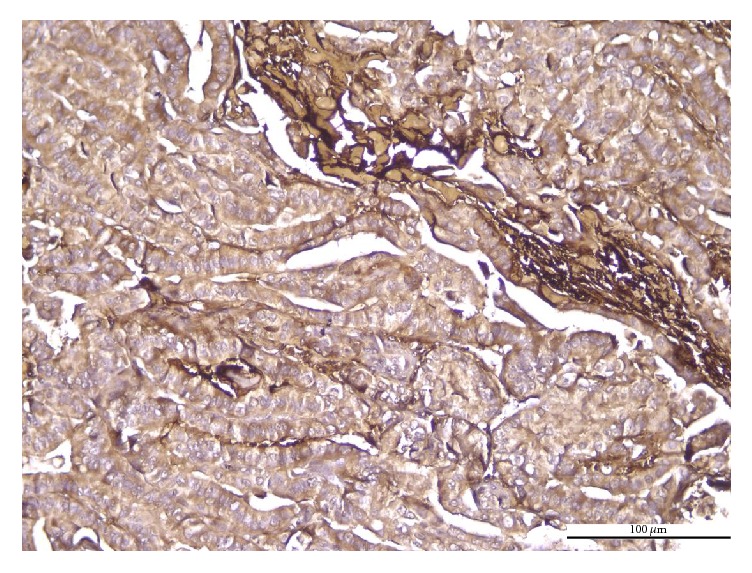
Tall cell PTC. Positive cytoplasmic PN, exhibiting focal apical and basal immunoreaction of moderate intensity; positive PN in intratumoral stroma (IHC anti-PN, ×200).

**Figure 4:**
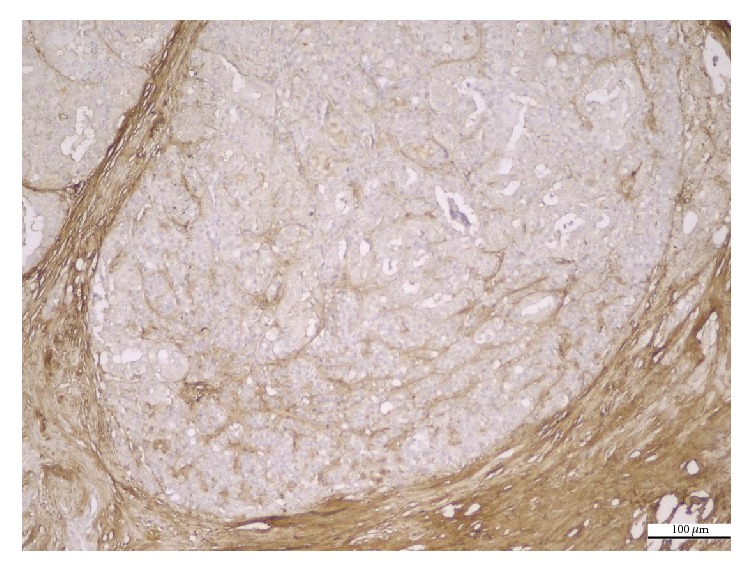
Oncocytic PTC. Negative PN expression in tumor epithelial cells and positive PN expression in tumor stroma (IHC anti-PN, ×200).

The histological variants of PTC showed different patterns of PN immunoreaction. The immunoexpression was diffusely cytoplasmic, with weak apical or basal polarization, in conventional (Figure 1), follicular, and macrofollicular (Figure 2) variants. The tall cell variant was characterized by localized immunoexpression, with predominantly apical distribution, along with focal infranuclear positivity (Figure 3). The immunoreaction was predominantly negative or very weak in oncocytic variant (Figure 4).

The intratumoral stromal PN expression was variable within the histological variants of PTC, from strong positivity in fibroblasts and collagen fibers up to lack of expression.

PN expression has been negative or weak, exhibiting a homogenous and diffuse cytoplasmic distribution in the follicular cells of nonneoplastic thyroid tissue.

#### 3.2.2. Semiquantitative Assessment

Tumor cells' PN expression has been evaluated as low score in 14 cases (28.0%) and with high score in 36 cases (72.0%) (Table 1). Intratumoral stroma exhibited PN negativity or weak expression in 16 cases (32.0%), whereas the other 34 cases (68%) showed PN strong positivity (Table 2).

#### 3.2.3. Correlations with Clinicopathological Prognostic Factors

The results of the statistical analysis between PN (low versus high expression) in tumor cells and clinicopathological features are summarized in Table 1. Statistically significant differences were registered only between PN immunoreaction and histological variants (*p* = 0.0002). A high PN score was more frequently noted in conventional subtype than in oncocytic subtype (OD = 105, CI 3.73–2948.28, *p* = 0.0062).

Table 2 synthesizes the correlation between PN stromal expression (negative versus positive) and clinicopathological features. Our results show significant differences between stromal PN immunoreaction and tumor stage (early versus advanced stages) (*p* = 0.04) and extrathyroidal extension (*p* = 0.008). Moreover, a high PN score was more frequently observed in advanced tumor stage (OR 0.28, 95% CI 0.07–0.99; *p* = 0.0491) and in the occurrence of extrathyroidal extension (OR 0.16, CI 0.03–0.67, *p* = 0.0124)

We have also noted a very close value to the statistical significant *p* value for the lymph node metastasis.

## 4. Discussion

PN is encoded by a gene located on chromosome 13 (13q13.3), in humans [55]. Structurally, it is formed by one N-terminal constant domain, one cysteine-rich domain (EMILIN-like), four fasciclin-repetitive-Fas domains, and one C-terminal hydrophilic domain exhibiting a variable structure according to the isoform [3, 4, 55].

Currently, eight PN isoforms are known, only five of them being sequenced and identified in different tissues: isoform 1 or (a) in osteosarcoma, isoform 2 or (b) in human placenta, isoform 3 or (c) in ovarian carcinoma, and 2 (b), 4 (d), and 5 (e) in either normal or tumoral urinary bladder [3, 8, 56–58].

Different PN isoforms may variably influence ECM fibrillogenesis [59] but it is still unknown if their effect on ECM increases the invasiveness or metastatic potential [13, 60, 61].

During the last 15 years, several papers provided evidences that support PN involvement in different malignancies. According to these studies, stromal PN expression is a negative prognostic factor for patients' survival [13, 28, 32, 41, 42] and, in association with epithelial PN, is significantly correlated with different clinicopathological prognostic factors [11, 13, 20, 35, 44, 47, 62, 63]. PN involvement in the epithelial-mesenchymal transition (EMT) has been also a matter of research interest, due to its potential therapeutic target value [8, 13, 39, 64–66]. Therefore, PN expression was analyzed in correlation with EMT (vimentin, elastin, and collagen) and angiogenesis specific markers, demonstrating its involvement as a promoter of this process [12, 15, 39, 63, 67].

Few papers addressed PN in thyroid tumors, predominantly using techniques of molecular biology (cDNA microarrays and real-time PCR) [58, 68–70]. Eight h-periostin isoforms have been identified in both thyroid carcinoma and in corresponding nonneoplastic tissues, all of them being related to thyroid carcinogenesis, invasion, or lymph node metastasis, regardless of differences between their expression pattern [58]. A high PN gene expression is associated with aggressive and poorly differentiated PTCs [68] and is correlated with specific morphological cellular features (loss of polarization and cohesiveness) registered in the invasive front of the tumor [69]. Only one of the four studies from literature has also analyzed PN immunoexpression, within a rather limited number of cases (10 normal thyroids, 10 follicular adenomas, 10 follicular thyroid carcinomas, and 10 PTCs samples, resp.) [70]. No PN staining has been noticed in normal thyroid tissue, in follicular adenoma, and in follicular thyroid carcinoma, and only 4 cases from a total of 10 PTCs showed a diffuse cytoplasmic immunoreaction [70].

Within this context, the present study provides new data regarding the specific PN immunoexpression in epithelial tumor cells and intratumoral stroma, in different histological subtypes of PTC.

To the best of our knowledge, this is the first report of qualitative differences in epithelial and stromal PN expression between conventional, follicular, macrofollicular, tall cell, and oncocytic subtypes. Thus, the idea that PN may be tissue-specific [11] is strengthened by supplementary evidences of its heterogeneity, reported in different histological subtypes of a specific tumor, such as clear cell, papillary, and chromophobe renal cell carcinoma types [33], and conventional and nonconventional osteosarcoma subtypes [71].

The pivotal role of PN synthesis in different malignancies is currently under scrutiny, by comparing the involvement of tumor epithelial cells with that of the tumor stromal component. As a consequence, it has been hypothesized that PN acts in a cell-type-dependent manner related to its expression in stromal versus epithelial cells, as a result of the activity of different PN terminal regions [13].

This hypothesis has been the starting point of our work which has individually quantified PN immunoexpression in tumor cells and in tumor stroma, respectively. We have additionally refined the reported scores already used for PN assessment [25, 53, 54], considering both the percentage of positive cells and the reaction intensity, using a threshold to label the investigated cases into low and high score categories. This modality of semiquantitative evaluation, based on a specific algorithm, has not been yet applied in thyroid tumor pathology.

Our study showed a heterogeneity of PN stromal immunoexpression, comparable to other malignancies reporting either PN positivity [19, 22, 23, 49, 64] or PN negativity [72]. The most papers have reported that stromal PN has a more aggressive potential than the epithelial PN. This aggressiveness can be attributed to the capacity of the PN produced by the stromal components to act not only by intracellular signaling pathways but also by its fibrillogenic potential within ECM, its C-terminal region interacting with ECM molecules [73, 74].

Our results support the dominant protumorigenic role of stromal PN, while epithelial PN action is less evident. We found that the high stromal PN expression is significantly associated with an advanced tumor stage and extrathyroidal extension. Similar results are also reported in renal cell carcinoma [31, 33], prostate [13, 27, 28], penile [75], and breast cancer [20, 23]. There are no available literature data about the stromal PN profile in thyroid tumors.

On the other hand, PN overexpression in tumor epithelial cells was correlated with specific histological PTC variants, the highest risk being registered for the conventional subtype in comparison to the oncocytic one. Our data are supplementing other results in the mainstream publications. Strictly referring to the thyroid pathology, the single published paper on PN immunoexpression in PTC [70] reports a correlation between PN overexpression and clinicopathological features (i.e., extrathyroidal invasion, distant metastasis, and higher grade staging).

Despite the small number of cases, the authors outline the correlation between PN, ETM, and an aggressive tumor behavior [70]. Moreover, they consider that PN could be a stronger negative prognostic marker than B-RAF, regardless of B-RAF mutation [70]. In other types of malignancies, comparable relationships are demonstrated in renal cell carcinoma (mainly for clear cell subtype) where a greater tumor epithelial PN expression is significantly associated with sarcomatoid differentiation, higher tumor stage, lymph node metastases, and poor overall survival [32, 33] and also in hepatocellular carcinoma, where PN correlates with microvascular invasion, multiple tumors, and advanced tumor stage [41, 42].

Taken together, our results are consistent with the complex framework of controversies regarding PN role in carcinogenesis, particularly for the thyroid location, and support the interest in understanding its relationship with different tumor behaviors. Further research is needed for the validation of PN current status as a promising biomarker.

## 5. Conclusions

Our study demonstrates a wide variability of PN expression in PTC, both in tumor epithelial component and in tumor stroma. High stromal rather than epithelial PN expression is associated with an aggressive tumor behavior. These results support PN involvement in tumor progression and its possible use as a prognostic marker.

## Figures and Tables

**Table 1:** PN expression in tumor epithelial cells and clinicopathological characteristics of PTC.

Clinicopathologic features	Case number		PN				*χ* ^2^	OR (95% CI)
			Low score		High score			
	#	%	#	%	#	%	*p value*	
*Sex*								
Female	41	82	12	29.27	29	70.73	*p* = 0.6699	0.69 (0.12–3.81)
Male	9	18	2	22.22	7	77.78		

*Age*								
<45	21	42	7	33.33	14	66.67	*p* = 0.4748	0.63 (0.18–2.20)
>45	29	58	7	24.14	22	75.86		

*Tumor stage*								
Stages I, II	24	48	7	29.17	17	70.83	*p* = 0.8599	0.89 (0.26–3.07)
Stages III, IV	26	52	7	26.92	19	73.08		

*Histologic subtype*								
Conventional	10	20	0	0	10	100	**p** = 0.0002	10.86 (0.55–211.91)
Follicular	21	42	7	33.33	14	66.67		
Macrofollicular	7	14	0	0	7	100		1.4 (0.02–78.80)
Tall cells	4	8	0	0	4	100		1.00 (0.24–4.13)
Oncocytic	8	16	7	87.5	1	12.5		105.00 (3.73–2948.28)

*Multifocality*								
Yes	34	68	10	29.41	24	70.59	*p* = 0.7459	0.8 (0.20–3.08)
No	16	32	4	25	12	75		

*Tumor size*								
<2.18 cm	35	70	10	28.57	25	71.43	*p* = 0.8907	0.90 (0.23–3.53)
>2.18 cm	15	30	4	26.67	11	73.33		

*Lymphovascular invasion*								
Absent	36	72	12	33.33	24	66.67	*p* = 0.1780	0.33 (0.06–1.73)
Present	14	28	2	14.29	12	85.71		

*Lymph node metastasis*								
Absent	43	86	12	27.91	31	72.09	*p* = 0.9710	1.03 (0.17–6.06)
Present	7	14	2	28.57	5	71.43		

*Extrathyroidal invasion*								
Absent	27	54	8	29.63	19	70.37	*p* = 0.7810	0.83 (0.24–2.90)
Present	23	46	6	26.09	17	73.91		

*χ* ^2^: chi-square test; OR: odd ratio; CI: confidence interval.

**Table 2:** PN expression in intratumor stroma and clinicopathological characteristics of PTC.

Clinicopathologic features	Case number		PN				*χ* ^2^	OR (95% CI)
			Low score		High score			
	#	%	#	%	#	%	*p value*	
*Sex*								
Female	41	82	13	31.71	28	68.29	*p* = 0.9246	1.07 (0.23–4.99)
Male	9	18	3	33.33	6	66.67		

*Age*								
<45	21	42	5	23.81	16	76.19	*p* = 0.2907	1.05 (0.55–6.84)
>45	29	58	11	37.93	18	62.07		

*Tumor stage*								
Stages I, II	24	48	11	45.83	13	54.17	**p** = 0.0439	0.28 (0.07–0.99)
Stages III, IV	26	52	5	19.23	21	80.77		

*Histologic subtype*								
Conventional	10	20	5	50	5	50	*p* = 0.7522	0.40 (0.08–1.90)
Follicular	21	42	6	28.57	15	71.43		
Macrofollicular	7	14	2	28.57	5	71.43		1.00 (0.15–6.64)
Tall cells	4	8	1	25	3	75		3.00 (0.22–39.60)
Oncocytic	8	16	2	25	6	75		1.20 (0.12–11.86)

*Multifocality*								
No	34	68	13	38.24	21	61.76	*p* = 0.1683	0.37 (0.08–1.56)
Yes	16	32	3	18.75	13	81.25		

*Tumor size*								
<2.18 cm	35	70	9	25.71	26	74.29	*p* = 0.1455	2.52 (0.71–8.96)
>2.18 cm	15	30	7	46.67	8	53.33		

*Lymphovascular invasion*								
Absent	36	72	13	36.11	23	63.89	*p* = 0.3176	0.48 (0.11–2.04)
Present	14	28	3	21.43	11	78.57		

*Lymph node metastasis*								
Absent	43	86	16	37.21	27	62.79	*p* = 0.0503	0.11 (0.006–2.07)
Present	7	14	0	0	7	100		

*Extrathyroidal invasion*								
Absent	27	54	13	48.15	14	51.85	**p** = 0.008	0.16 (0.03–0.67)
Present	23	46	3	13.04	20	86.96		

*χ* ^2^: chi-square test; OR: odd ratio; CI: confidence interval.
